# Unlocking the computational design of metal–organic cages

**DOI:** 10.1039/d2cc00532h

**Published:** 2022-02-25

**Authors:** Andrew Tarzia, Kim E. Jelfs

**Affiliations:** Department of Chemistry, Molecular Sciences Research Hub, Imperial College London White City Campus, Wood Lane London W12 0BZ UK k.jelfs@imperial.ac.uk

## Abstract

Metal–organic cages are macrocyclic structures that can possess an intrinsic void that can hold molecules for encapsulation, adsorption, sensing, and catalysis applications. As metal–organic cages may be comprised from nearly any combination of organic and metal-containing components, cages can form with diverse shapes and sizes, allowing for tuning toward targeted properties. Therefore, their near-infinite design space is almost impossible to explore through experimentation alone and computational design can play a crucial role in exploring new systems. Although high-throughput computational design and screening workflows have long been known as powerful tools in drug and materials discovery, their application in exploring metal–organic cages is more recent. We show examples of structure prediction and host–guest/catalytic property evaluation of metal–organic cages. These examples are facilitated by advances in methods that handle metal-containing systems with improved accuracy and are the beginning of the development of automated cage design workflows. We finally outline a scope for how high-throughput computational methods can assist and drive experimental decisions as the field pushes toward functional and complex metal–organic cages. In particular, we highlight the importance of considering realistic, flexible systems.

## Abstract 2

Metal–organic cages are macrocyclic structures that can possess an intrinsic void for application in encapsulation, sensing and catalysis. In this article, we highlight approaches and limitations to their computational design.

## Graphical abstract

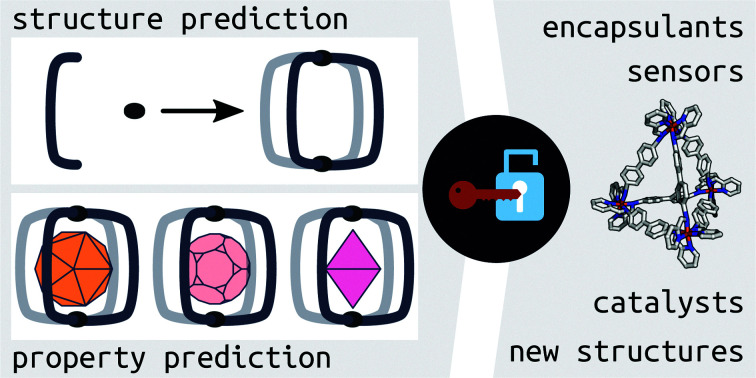

## 1 Introduction

Porous materials have been the focus of significant development in recent decades. These materials are chemically diverse and include extended and molecular structures in the solid and solution state. ‘Hybrid’ materials are a subset of porous materials that contain a combination of metal and organic components, and include the well-known metal–organic frameworks (MOFs) and, the focus of this review, metal–organic cages (MOCs; equivalent to metal–organic polyhedra (MOPs) and supramolecular coordination cages (SCCs)). MOCs are self-assembled, hybrid, macrocyclic structures that form (supra-)molecular architectures typically containing an intrinsic void; this fits with the IUPAC definition of a molecular cage as a “polycyclic compound having the shape of a cage”.^1^ The intrinsic void of MOCs leads to their potential applications^2,3^ in encapsulation,^4^ drug delivery,^5^ enzyme mimicry and catalysis,^6–8^ sensing and separations,^9^ and in soft materials,^10,11^ making them attractive candidates for biological and membrane applications.

MOCs are typically synthesised through a self-assembly process, where components undergo reversible reactions towards a thermodynamic equilibrium. This leads to the expectation that the major product will often be the thermodynamic product, assuming no kinetic traps, and that design principles can be used to rationally target specific self-assembly outcomes.^12^ In most cases, chemists have side-stepped some complexities of MOC formation by focusing on rigid and symmetrical building blocks, metals with well-defined geometries, and the design principles borne from those choices. However, there has been a drive to increase MOC complexity and potential function through the self-sorting of multiple ligands into heteroleptic cages, unsymmetrical ligands into asymmetrical MOCs, or ligands with secondary functions into functional materials.^13–15^ By doing so, the degrees of freedom in MOC design increases significantly and becomes difficult to do *via* intuition.

Although computational modelling can provide atomistic or electronic-level insights into MOCs that are not directly experimentally accessible, MOCs have received limited computational research effort when compared to other porous materials such as porous organic materials^16^ and MOFs.^17,18^ We will review how computational tools can be applied to the study and design of MOCs. Throughout materials science and drug discovery, the application of computer-driven approaches has grown significantly. To rationally design any material from scratch is a multi-variable complex problem. Broadly speaking, there are multiple ways to tackle this problem that we simplify to two approaches:^19^ (i) a conventional approach starts with selected precursors, examines the products and their properties to find the optimal material, (ii) an inverse approach designs the material (and eventual precursors) starting from a specific property. Regardless of which approach is chosen, both benefit from integrated feedback between experimental and computational research.^20^ While, computational methods can complement experimental design choices in MOCs, they range in cost and difficulty/complexity. Here, we focus on low-cost or high-throughput approaches that afford a large coverage of chemical space.

**Fig. 1:**
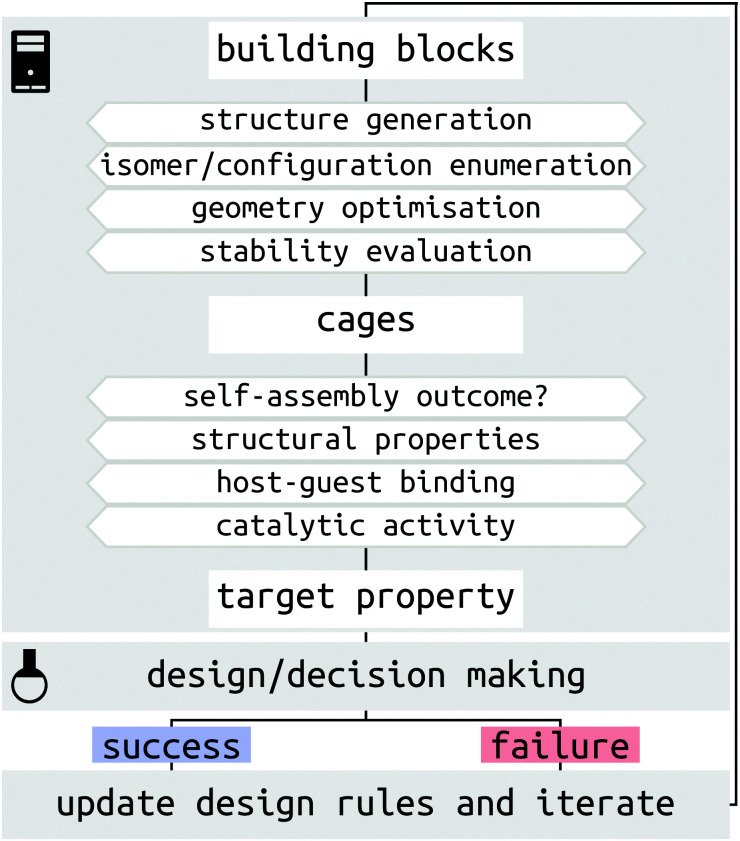
Schematic of the steps and challenges toward MOC design and assisting experimental decision making. The broader process of MOC design will be an iterative consideration of successes and failures, which update our design principles.

There are several challenges for the computer-driven design of MOCs (Fig. 1). One of the main challenges is structure prediction, which corresponds to predicting the molecular configuration(s) of the self-assembled product(s) from building blocks (organic and metal components). The prediction of solid-state structures of MOCs represents an extremely challenging crystal-structure prediction problem that is yet to be tackled. Although the prediction of solid-state structures of similar, porous organic, molecules has been accomplished,^21,22^ the introduction of many metal-centres increases the cost and difficulty of these calculations. Further to this, many challenges relate to the issue of the cost and complexity of modelling systems with multiple metals at either the density functional theory (DFT) or classical force field (FF) level. Finally, the introduction of dynamics, including solvent, and complex interactions in the study of host–guest systems poses a great challenge for high-throughput computation and property prediction.

Significant progress in both software and hardware has made the advancements we highlight below possible. As we show, it is now possible to evaluate orders of magnitude more MOC candidates *in silico* than in the lab and in a shorter time. We outline this article based on a description of MOC structures and recent solutions to the challenges of studying metal-containing systems (Section 2), an introduction of structure generation processes (Section 3), methods for predicting the outcomes and processes of MOC self-assembly (Sections 4 and 5), examples of evaluating MOC host–guest properties (Section 6), and an outlook into the future of MOC design (Section 7).

## 2 Describing metal–organic cage structures

The modular approach to MOC synthesis from combining metal-containing and organic building blocks allows for their near-infinite tunability. Indeed, MOCs come in a vast array of shapes and sizes and those structures are directly related to their properties. There are several reviews that cover the structural diversity and design principles of MOCs.^23–35^ MOCs are often described using *M*_*n*_*L*_*m*_ nomenclature, where *M* and *L* are the metal and organic building block(s), respectively, and *n* and *m* are their respective stoichiometries. Hay and co-workers developed a similar nomenclature for MOCs^36^ that describes a topology defined by the number of vertices, edges and faces. These nomenclature do not directly provide topological or geometrical information, or direct information on the building block connectivity. However, prevalent structures in the literature are often linked with certain topologies and geometries, which is useful for the discussion and design of MOCs based on these templates. Design principles for targeting specific topologies/templates provide the experimental and computational chemist with a good starting prediction.

**Fig. 2:**
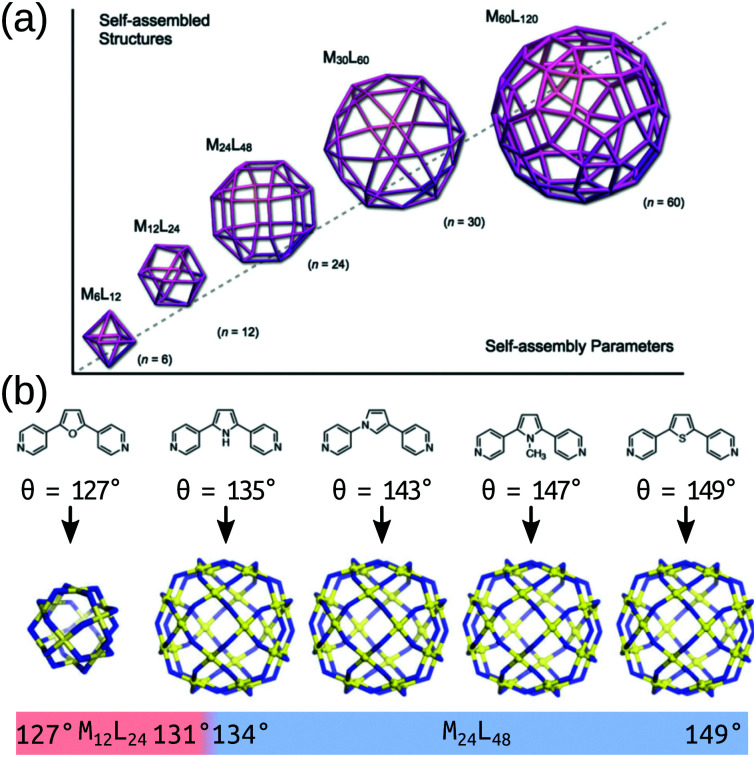
(a) A schematic of the structure relationships observed in a family of roughly spherical coordination polyhedra with general formula *M*_*n*_*L*_2*n*_, where metals (*M*) and bridging bis(pyridine) ligands (*L*) are mapped onto the respective vertices and edges of the polyhedra. (b) Critical structural switch between Pd_12_L_24_ and Pd_24_L_48_ structures in a chemically well-defined system as a function of ligand bite angle. Reprinted with permission from ref. 40.

In its simplest form, MOC design stems from understanding the preferred geometry around a metal atom and installing organic components that link multiple metal atoms based upon this.^37^ The reticular chemistry approach that is so beneficial to the MOF field^38^ applies to MOCs as well.^25^ Many of the early advancements in MOC design stem from the assumption that MOC building blocks are rigid, which allows for the use of the “directional bonding” approach (also termed the “ligand-directed” or “symmetry-interaction” approaches). In this approach, the relationship between the bonding vectors of a metal–ligand interaction define the geometry of the connection between building blocks. Therefore, two building blocks with well-defined bonding vectors can be mapped onto idealised MOC structures.^37,39^ Using rigid components and targeting concave structures has lead to the natural exploitation of high-symmetry geometries (polygons, polyhedra) over the last two decades.^24^ Fitting with the directional bonding approach, topological selection based on the “bite angle” of the chosen ligand has been crucial in the development of MOC structures. This design principle has been explored in detail by Fujita and co-workers over many articles looking at Pd-based systems^40^ and builds on the geometrical constraints of the building blocks to determine a favourable topology (Fig. 2). This work shows that the bite angle rule applies generally and has led to the robust development of large homoleptic and heteroleptic MOCs with multiple metal species.^29,41–45^

Even with these design rules in hand, deviations from expectation occur.^12,36^ This is especially true when ligands deviate, even slightly, from the rigid approximation. For example, a review by Young *et al.*^36^ focused on rigid building blocks found that there were many deviations from expectation due to small variations in the preferred geometry of building blocks. Targeting MOCs that mimic the fidelity of enzymes through adaptive or stimuli responsive behaviour requires the introduction of flexibility. There is flexibility at the building block-level, which impacts the self-assembly of MOCs, and flexibility at the cage-level, which impacts MOC properties. Conformationally flexible ligands decrease the preorganisation in a system, which can result in unexpected topological outcomes and a knock-on effect on properties.^46–49^ Computational design of systems with flexible ligands is an additional challenge because it increases the degrees of freedom in the structure/property prediction.

Modelling and designing MOCs is inherently limited by our capability to model large structures (100s of atoms) with multiple metal-centres. There are a wealth of classical and quantum-mechanical computational approaches for modelling metal-centres^50,51^ and MOFs^17,18,52–54^ that may be applicable to MOC systems. There have been recent advances in handling the diversity of metal complexes in a high-throughput fashion. However, the balance of cost *vs.* accuracy and the need for expert knowledge to properly consider complex electronic properties for different metal species remains a barrier to usage in design workflows. In particular, transition metals present a challenge for DFT methods.^55^ Part of the challenge of initiating a study of metal-containing systems is whether the chemistry of interest has been implemented, for example does the available software include a FF with the parameters required for the specific metal being modelled, and/or can that system be accurately modelled with readily available techniques. General-purpose FFs ensure this for many metal geometries. For example the Universal Force Field (UFF),^56^ and the associated extension for MOFs,^57,58^ and GFN-FF,^59^ in software such as *xtb*,^60^ Open Babel^61^, Avogadro^62^ and the General Utility Lattice Program (GULP).^63,64^ In the case of a FF not containing parameters for the metal of interest, it is common to parameterise FF parameters, which is facilitated by software such as the Metal Center Parameter Builder.^65^ Similarly, transferable dummy atom models^66,67^ are approaches to modelling the geometry of metal centres without needing to explicitly describe the bonded interactions between the metal and ligand. In a dummy-atom approach, the metal–ligand interactions are represented as electrostatic interactions between charged dummy atoms (placed around the metal atom in a rigid geometry) and the ligands.

Another approach is generalised energy-based fragmentation (where systems are rationally fragmented into smaller components), which was recently shown to allow the study of large supramolecular systems at much lower cost than modelling the full system.^68^ Finally, the freely-available GFN*n* semiempirical density-functional tight-binding methods available in the *xtb* software (herein, termed GFN*n*-xTB) and GFN-FF method represent easy-to-use and broadly applicable, low-cost solutions to modelling metal-containing species.^59,60,69^ This family of methods were parameterised for large parts of the periodic table (*Z* ≤ 86), and have been shown to be robust for geometries, frequencies and noncovalent interactions of metal-containing species (including MOCs).^70^ Similarly, advances in robust and low-cost composite DFT methods (*e.g.*, B97-3c and r^2^SCAN-3c)^71,72^ provide technically accessible methods applicable to metal-containing structures^73^ that can reproduce geometries and energetic rankings.

The chemical space available to MOC chemists is vast. On top of the very large chemical space available to organic ligand design, many different metals have been used in experimental studies (see ref. 74). When evaluating approaches to modelling metal-containing systems, the diversity in electronic configuration, spin state, oxidation state and bonding/geometry presents issues in finding a “best-practice” or universal solution.^50,75^ It is possible to focus on a single “well behaving” metal and on exploring the ligand chemical space. However, this approach is fundamentally limiting. There have been recent advances in exploring transition-metal chemical space by developing cheminformatic-inspired approaches.^75,76^ Overall, recent literature, including our own work, has shown significant progress in user-friendly, high-throughput approaches for tackling metal-containing systems.

## 3 Approaches to computational structure generation of metallo-molecular architectures

There are two main approaches to single-molecule structure generation of MOCs: top-down or bottom-up (*de novo*). In top-down approaches, molecules are built by placing building blocks on a template, while bottom-up approaches, commonly used in drug design, build molecules by fitting fragments into some target geometry. A common issue for both approaches that the user must be aware of is that arbitrarily combining components can diminish synthesisability.^77,78^ The top-down approach benefits from being based on templates, which inherently encodes design rules into the process and the building blocks used.

### 3.1 Bottom-up or *de novo* structure generation

**Fig. 3:**
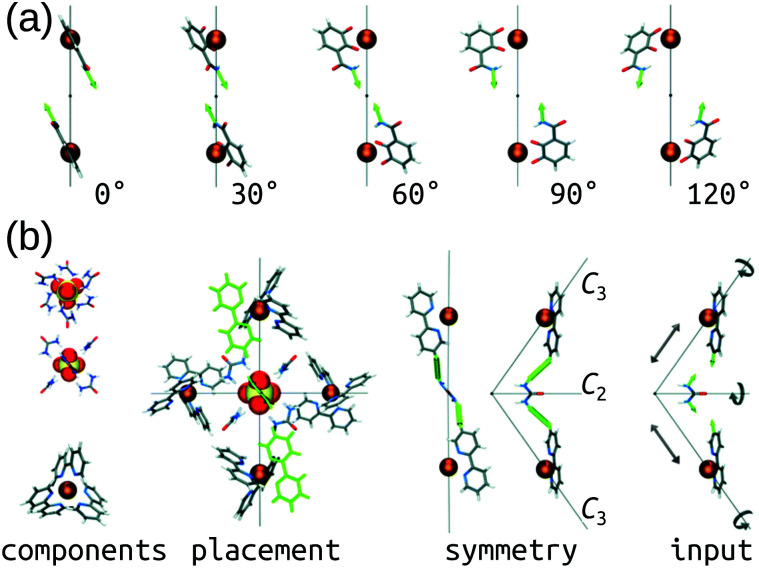
(a) Vector poses for the input fragment obtained by vertex rotation about the *C*_3_ axis. Amount each vertex is rotated from the initial position is given below each structure. (b) Generation of *HostDesigner* starting structure. (left) Structure of the [SO_4_(urea)_6_]^2−^ complex, viewed down the *C*_3_ (top) and *C*_2_ (bottom) axes, and of the [Ni(bpy)_3_]^2+^ complex, taken from crystal structure coordinates (CSD REFCODE: CUHVUW). (middle-left) Placement of [SO_4_(urea)_6_]^2−^ and [Ni(bpy)_3_]^2+^ components into a *T* symmetric assembly. (middle-right) Design target is an edge molecule illustrated with generic linkages (green cylinders) between the bpy and urea groups. (right) Input fragment, with geometric drives depicted by grey arrows. Reprinted with permission from ref. 36.

The Hay group developed a bottom-up, *de novo* approach to transition-metal complex and cage structure generation that moves away from the limitations of idealised geometries and bond-vector approaches.^36^ Their approach is implemented in their free software, *HostDesigner*,^79,80^ which has been used for designing metal-ion binders to MOCs.^77,81–87^ Their structure-based algorithms work by docking “linking fragments” (from a library) between the target “complex fragments”, then covalently connecting those fragments, which produces new molecules in active conformations. All possible connection points, conformations, linking isomers and stereoisomers are explored in the assembly algorithm (*e.g.*, the rotations and translations of the two fragments in Fig. 3a). Fig. 3b shows a MOC construction workflow starting from two complex fragments. In *HostDesigner*, Hay and co-workers define a fitness function for the *de novo* generation of a host based on the fit between bonding vectors and whether steric clashes occur. Recent iterations use a scoring function that considers multiple properties: (1) binding energy with the guest, (2) conformational strain and (3) entropic cost to binding, all of which can be evaluated with FF calculations or empirically.^77^ They show the utility of this approach in the design of *M*_4_*L*_6_ MOCs^88^ and ion-pair *ML*_3_*A* helicates (*A* is an anion).^89^ Custelcean *et al.* designed a new Ni_4_L_6_ sulfate receptor (Fig. 3(b)), which was the first example of the design of a MOC lined with urea binding sites.^88^ From their top candidates, they selected and successfully synthesised one that outperforms other synthetic sulfate receptors. For a tutorial review into using *HostDesigner*, see ref. 84 and the documentation with the software.

### 3.2 Top-down structure generation

**Fig. 4:**
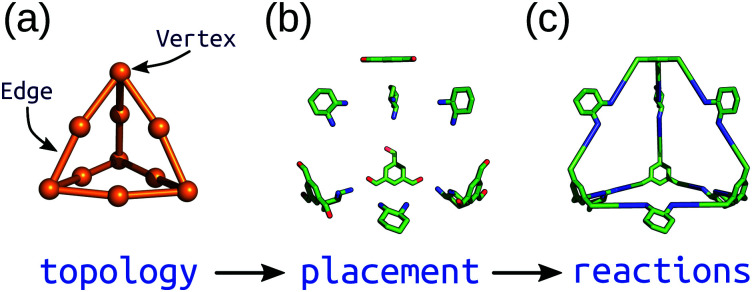
Schematic of the construction process of an organic cage in *stk* starting from a (a) topology graph of vertices connected by edges. The supplied building blocks are (b) placed and aligned on the topology, and then (c) “reactions” are performed between them. Reprinted from ref. 95, with the permission of AIP Publishing.

Top-down methods, where building blocks are placed on predefined geometries or topologies, are often used in the generation of solid-state porous materials.^90–94^ Such approaches benefit from the starting points provided by existing design principles and structures. We have implemented a topology-based approach in our software, *stk* (https://github.com/lukasturcani/stk), which we recently improved to handle metal–organic systems.^95^*stk* is an open-source, Python toolkit that provides modular, easy-to-use functionality for generating chemical structures by placing building blocks on a “topology graph”. A topology graph defines an idealised geometry of “vertices” and “edges” (edges connect vertices), where the vertices and edges define independent alignment and reaction processes to perform on building blocks (which are placed on vertices) during construction. This approach is well suited to the descriptions of MOC structures described above. Fig. 4 shows a simplified workflow of *stk* construction. Some crude, rigid body optimisation protocols are available within *stk*, but in general, the initially constructed *stk* molecular models require further optimisation. For this, we have developed the *stko* package (https://github.com/JelfsMaterialsGroup/stko)^96^ that takes in *stk* molecules and can perform property evaluations (including single-point energy calculations, geometrical analysis) and geometry optimisations. Currently, *stko* includes wrappers for RDKit,^97^ GULP,^63,64^ Macromodel^98^ and *xtb*.^60,69^ We have used GULP and *xtb* in our workflows, which allow for conformer searches and geometry optimisations of MOCs.

**Fig. 5:**
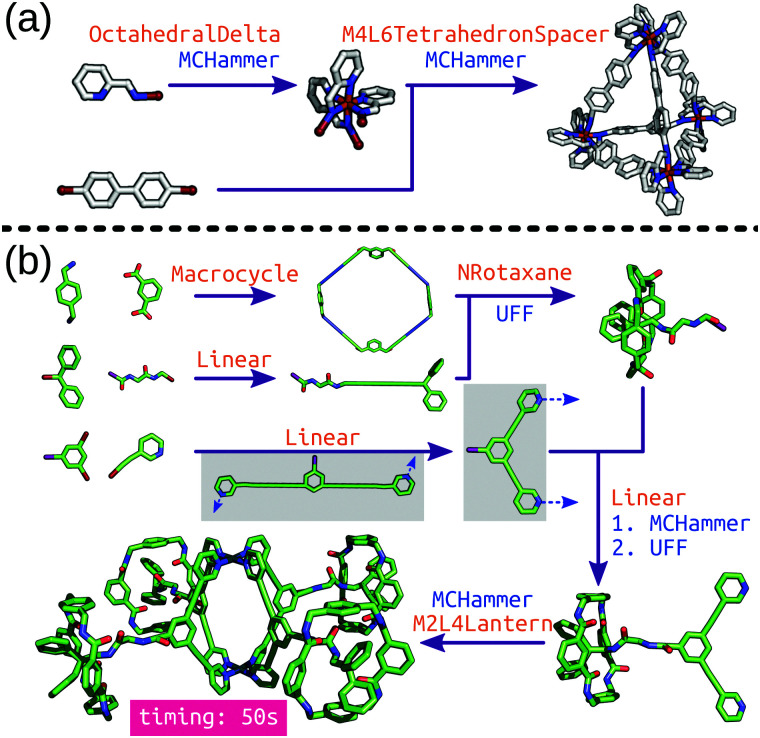
Hierarchical construction (a) of a Δ-*M*_4_*L*_6_ cage from an octahedral complex (this code was taken directly from the *stk* documentation) and (b) of a metallo-[5]rotaxane from ref. 100. Each purple arrow is a construction process, orange text is the topology graph used and blue text is the optimisation process used. The grey boxes show the (left) initial structure generated by *stk*, which must undergo conformer sampling to find the (right) conformer used for cage construction. MCHammer is a low-cost geometry optimisation function implemented in *stk*; further optimisation would be required.

*stk* can construct many different molecule classes beyond cages, including polymers, macrocycles, metal complexes, rotaxanes, host–guest complexes and extended porous materials (COFs and MOFs). One of the key features is the transferability of *stk* molecules, where a constructed molecule can then be used as a building block in the construction of a new molecule in a hierarchical process. Importantly, *stk* is “chemistry agnostic” in the sense that any building block can be placed on any topology graph. Furthermore, *stk* can construct cages with arbitrary ligand placements and alignments, allowing for heteroleptic cage construction and the consideration of configurational isomers.^99^ This feature is especially crucial for MOC construction because the metal complex often needs to be constructed prior to cage construction (Fig. 5a) with a specific stereochemistry. Fig. 5b shows hierarchical model construction of a Pd_2_L_4_ cage with rotaxane-based ligands (code available at https://github.com/andrewtarzia/stk-examples).^100^ Finally, we updated the in-built optimisation options in *stk* to include a host–guest conformer generator that can very quickly optimise the position and orientation of guest(s) within a host.

**Fig. 6:**
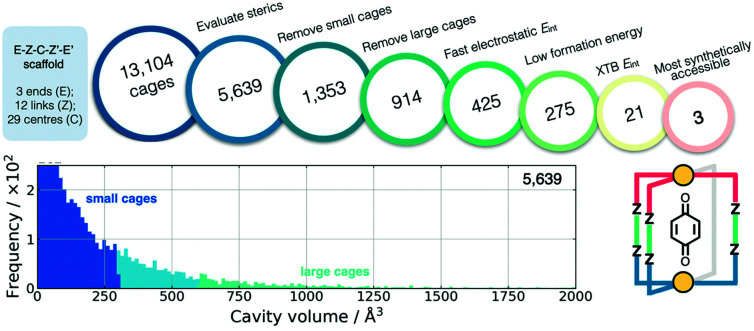
Filtering process used to find the three optimal hosts for benzoquinone based on simple geometric criteria, tight-binding DFT calculations, and synthetic accessibility of the constituent linker molecules. Adapted with permission from ref. 101. Copyright 2020 American Chemical Society.

Also using a top-down approach, Young *et al.* developed *cgbind* (available at https://github.com/duartegroup/cgbind), an open-source Python toolkit for the construction and evaluation of MOCs (a web-app is also available at http://cgbind.chem.ox.ac.uk).^101^ They show that *cgbind* generates structures close to crystal structure conformations without any external software, which is a huge benefit to high-throughput screening. *cgbind* also provides a series of analysis algorithms and can generate host–guest complexes. In a comparison to literature binding data, they show good performance of their fast binding-energy evaluators compared to semiempirical methods for a series of MOCs and guests. Fig. 6 shows a workflow for MOC screening they implement using *cgbind*, where ligands are generated in a combinatorial fashion based on “end”-“link”-“center”-“link”-“end” structures. They screen the possible 13104 Pd_2_L_4_ cages based on the cage geometry, the size of the cage, the host–guest binding energy (based on a quinone guest), the formation energy of the cage (calculated using GFN*n*-xTB) and, finally, the synthetic accessibility^102^ of the ligand.

### 3.3 Precursor generation

The generalisability of top-down approaches means that vast precursor libraries can be easily explored (*e.g.*, Fig. 5(b) and 6). However, the generation of transition metal complex structures is a developing field in itself and generating precursor building blocks of metal complexes is not trivial. We have introduced common supramolecular metal geometries into *stk* (*e.g.*, octahedral; Fig. 5a).^95^ There are also multiple software for generating 3D coordinates of transition metal complexes and to perform *de novo* generation^103^ of metal complexes: DENOPTIM,^104^*molSimplify*^105^ and *Molassembler*.^106^ For the organic components, free software like *RDKit*^97,107,108^ or Open Babel^61^ can easily generate 3D conformers from 2D representations of molecules. Rational precursor selection should be an integral part of material design to avoid synthesisability issues.^78^

## 4 Predicting topology and configuration of metal–organic cages

Much of the computational effort spent on MOC studies, so far, has been on trying to determine or rationalise the outcome of a self-assembly process. Assuming the thermodynamic product is formed, the connectivity of the building blocks and their stoichiometric ratios define the possible self-assembly outcomes (topologies). For example, mixing square-planar Pd(ii) (*M*) and ditopic N-donor ligands (*L*) in a 1 : 2 ratio should, under thermodynamic conditions, produces a *M*_*n*_*L*_2*n*_ topology. Still, predicting which topology forms from precursors remains a challenge as the topological preference (and/or the self-assembly process) is sensitive to solvent,^109^ counter/co-ions,^110^ and building block geometry and flexibility. To identify the preferred structure for an arbitrary ligand (or set of ligands), it is possible to directly calculate the relative energy of all possible topologies. However, the energy differences between configurations are often small and, for example, competing ligand–ligand interactions are on the same energy scale as those driving MOC self-assembly. Therefore, any approach would need to be sufficiently accurate to distinguish between the multiple low-lying energetic states while also being sufficiently efficient to be practical for prediction. Further complicating this issue is when multiple self-assembled structures are present in equilibrium or can be selected for by changing the environment, highlighting their closeness in relative energy and difficulty in distinction.^111^ A specific example of this problem is the “triangle *vs.* square equilibria” of Pd-based polygons,^112^ where the triangle is entropically favoured and the square is enthalpically favoured; the triangle becomes enthalpically favoured as the ligand is made more flexible.^113^ Uehara *et al.* explored the factors controlling the formation of Pd-based square and triangle structures based on a series of bridging ligands.^113^ They showed that DFT calculations considering implicit solvation can reproduce the effect on the equilibrium of steric crowding, bridging ligand length and solvent effects.

**Fig. 7:**
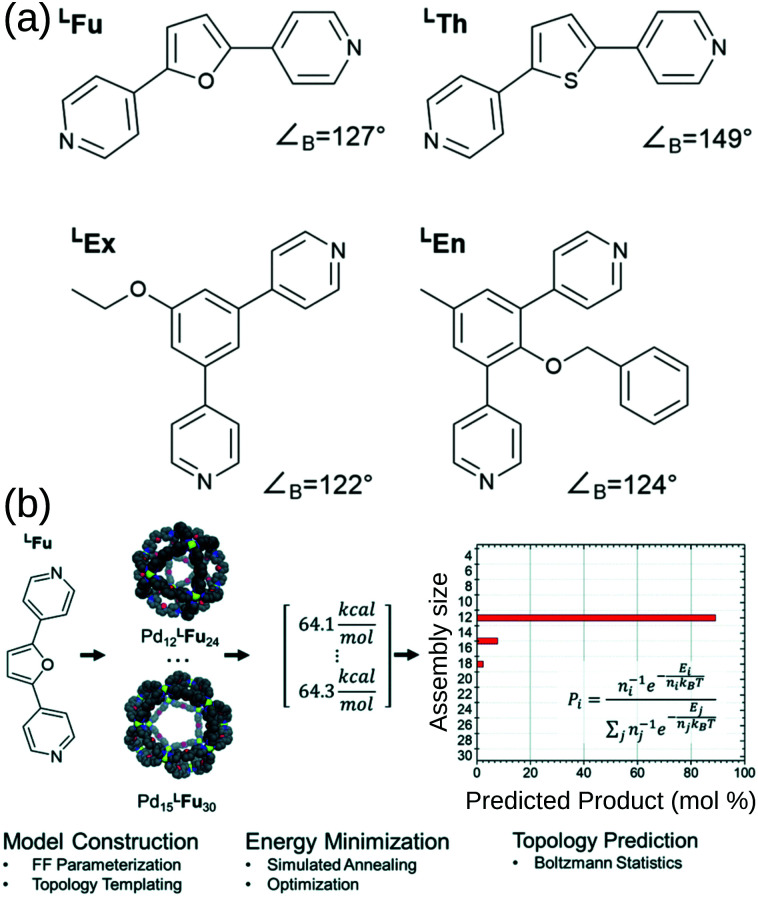
(a) Structures of the bipyridyl linker molecules used in this study. Bend angles, ∠_B_, are estimated from B3LYP/def2-TZV minimized structures. (b) Flow chart for topological prediction of homoleptic assemblies featuring the formation of homoleptic Pd_*x*_^L^Fu_2*x*_ assemblies. The linker structure is used to construct a library of possible assembly outcomes, which are subjected to a simulated annealing and structural optimization procedure using implicit solvation. The resulting minimum energies, *E*, are treated as microstates, and the topological distribution is determined using Boltzmann statistics with a weighting factor ‘*n*’ corresponding to the number of linker components in the assembly. Reprinted with permission from ref. 114.

Poole *et al.* developed a FF and structure/topology prediction workflow for Pd_*n*_L_2*n*_ (*n*: 3–30) structures.^114^ They predict the topologies of cages self-assembled from four different ligands (Fig. 7a). Given an FF that accurately reproduces cage free energies, they calculated the relative energy and Boltzmann statistical weight of each topology to make their prediction of the most likely topology (Fig. 7b). For ligands ^L^Fu and ^L^Th, they reproduce the major topology preference and uncover the presence of new or intermediate species, which they support with their own experimental work. Considering the more complex case of heteroleptic systems, they reproduce the experimentally confirmed critical concentration of ligands ^L^Fu and ^L^Th at which a Pd_24_L_48_ topology becomes preferred over the Pd_12_L_24_ topology. For ligands ^L^Ex and ^L^En, they reproduce the effect of endohedral functionalisation in favouring larger species than their bite angle would suggest; this result was supported by experiments. Their work reproduces and expands upon experimental data and shows that by providing atomistic insight into intermediate topologies and ligand dynamics, they can strengthen design principles toward rational design of MOCs. Indeed, they used this approach to help guide experimental control of the self-assembly of platinum architectures.^47,115^ However, the *a priori* knowledge of possible topologies and the number and cost of simulations remain barriers for this approach.

**Fig. 8:**
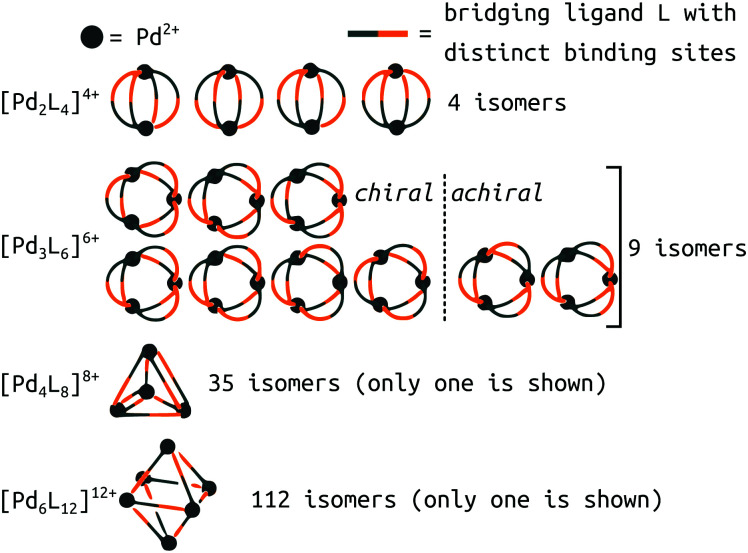
Potential isomers for metal–ligand assemblies of the general formula [Pd_*n*_L_2*n*_]^2*n*+^ (*n* = 2, 3, 4, or 6). The isomers differ in the relative orientation of the bridging ligands L, which have two distinct binding sites. The number of isomers increases from 9 → 16, 35 → 68, and 112 → 186 if enantiomers are considered as well. Adapted from ref. 119.

A new level of complexity comes into play when considering the self-sorting^116^ of configurational isomers^14^ or stereoisomers.^117,118^ While design rules can guide structural predictions, unexpected behaviour often occurs, resulting in fascinating MOCs.^12^ Therefore, direct structure prediction, without the bias of predetermined outcomes, is necessary for future property prediction and design processes. Unsymmetrical ligands, for example, introduce complexity because multiple isomers can form during the self-assembly process. Recent work with unsymmetrical ligands highlights the combinatorial explosion that can occur when forming higher nuclearity topologies (Fig. 8).^119,120^ For example, there are 112 possible isomers for Pd_6_L_12_ structures that would need to be evaluated per ligand. Li *et al.* suggest that geometrical matching can rationalise self-sorting in their complex system. In our work,^121^ steric, geometrical and a mixture of steric and geometrical control is introduced to rationally favour a specific isomer. Studies like the above begin the path toward design rules for asymmetrical MOCs. We,^121^ and Yu *et al.*^120^ supported our findings with DFT calculations. However, applying similar methods to higher nuclearity species with many more isomers would be very expensive.

**Fig. 9:**
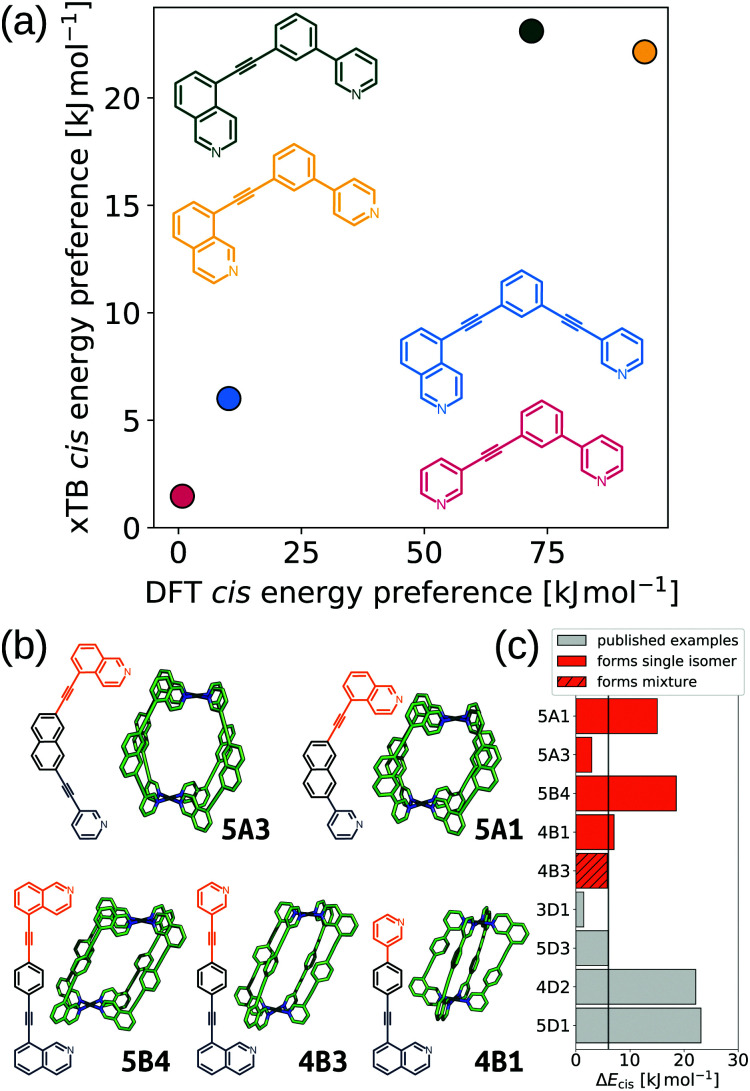
(a) Comparison of GFN2-xTB (DMSO) and DFT (PBE0/def2-SVP/D3BJ/CPCM(DMSO)) energy difference between the *cis* and next most stable isomer of cages formed in ref. 121 from the ligands 3D1 (crimson), 4D2 (yellow), 5D1 (dark green) and 5D3 (blue). (b) GFN2-xTB optimised structure of the *cis* isomer of selected cage ligands (hydrogen atoms omitted; C green, N blue, Pd cyan). Cage ligands are shown next to each structure with orange and navy indicating inequivalent ligand fragments. (c) *cis* isomer GFN2-xTB (DMSO) stability (Δ*E*_*cis*_) for all published^121^ and newly selected ligands (patterns distinguish their self-assembly outcomes). Adapted from ref. 99.

Furthering our exploration of these systems, we implemented a joint computational and experimental evaluation workflow for Pd_2_L_4_ MOCs formed from unsymmetrical ligands.^99^ In this example, we assumed a single topology (Pd_2_L_4_) was most likely to form based on enthalpic arguments. We targeted a low-cost, “good enough”, prediction of the self-assembly outcome to guide experimental decision making. Searching for ways to lower the cost, we found that the predictions of *cis* isomer preference by GFN2-xTB^69^ agreed with DFT and could reasonably predict whether an unsymmetrical ligand would self-sort into a single of four possible isomers (Fig. 9a). This allowed us to use a workflow based on FF and semiempirical methods for cage conformer searches, geometry optimisation and relative energy evaluation, resulting in structure generation in hours instead of days. We used *stk*^95^ to construct 60 unsymmetrical ligands and their respective cage isomers (four per ligand, 240 cages in total) from a series of hand-picked ligand building blocks. When coupled with geometrical heuristics of MOC stability (*e.g.*, “how square-planar is the metal centre?”), we ranked candidate ligands for their likelihood of forming only a single isomer in solution. Fig. 9b and c shows the five cages evaluated experimentally, and their respective *cis* isomer preference. Our predictions of self-sorting failed in at least one instance (4B3 did not self-assemble into a single isomer based on experimental validation). However, we purposefully tested around the previously found threshold for *cis* isomer preference to further strengthen our design principles. Additionally, our approach is general and counteracts the increased computational effort expected for more complex systems.

In summary, the structure prediction of MOCs can require a large number of calculations due to the configurational freedom. Overall, it is likely necessary to consider a very large number of possible topologies/isomers when predicting MOC structures. Using design rules and simplified models can make MOC structure prediction more tractable by narrowing the available search space and decreasing the cost of calculations. In an example approach, Yoshida *et al.* developed an effective model Hamiltonian that can help make MOC topology evaluation more efficient.^122^ However, to develop and apply low-cost methods requires robust data to build from, which emphasises the need for experimental and computational collaboration.

## 5 Understanding the self-assembly process

The self-assembly process of MOCs corresponds to a complex landscape of potential intermediates that, remarkably, produces a single species in many cases. This is the result of the large enthalpy gains designed into the systems that counter the entropy losses of forming ordered structures.^123^ By understanding the self-assembly process one can access and evaluate the potential intermediates *via* methods like above. On this path, substantial work done by Hiraoka and co-workers in quantitative (QASAP) and numerical (NASAP) analysis of self-assembly processes, with a specific focus on Pd and Pt-based MOC assembly,^124,125^ provides a map of complex cage-assembly processes. Their numerical, reaction-network analysis can reproduce experimental intermediate populations and uncover kinetically trapped species.^126–128^ Another approach is to directly model the self-assembly process. Yoneya *et al.* developed an MD simulation protocol for modelling the self-assembly of Pd-based MOCs (Pd_6_L_8_^129^ and Pd_12_L_24_^130^ cages). They implement a nonbonded dummy atom model^66,67^ to represent the metal atoms and maintain bond reversibility, and a coarse-grained (CG) solvent model that allows for cage assembly to be observed on MD time scales. Under the right conditions (determined through trial-and-error), they are able to explore the stages of MOC self-assembly in atomistic detail, providing further understanding and uncovering possible intermediates. While studying Pd_12_L_24_ assembly,^130^ they found that kinetic trapping occurs at smaller-sized clusters (*e.g.*, Pd_6_L_12_, Pd_8_L_16_, and Pd_9_L_18_), which can be decreased by increasing ligand bite angle (reproducing experimental findings). This model has been applied to study the assembly and stability of Pd-based MOCs,^131–133^ Hg-Based MOCs^134^ and MOFs.^135^ This approach is fundamentally generalisable because the dummy-atom approach is applicable to other metals.^66,67,136,137^

## 6 Exploring host–guest systems and confinement in metal–organic cages

The main applications of MOCs stem from their solid-state or solution-phase interactions with guest molecules to form host–guest complexes. How to compute the relevant properties for these applications will be discussed next. While the systems explored below are relatively simple host–guest complexes, very complex (*e.g.* multiple guest) complexes are experimentally viable and of interest to the community due to there applications in catalysis and sensing.^4^ There are multiple barriers (balancing cost and accuracy) to the computational design of host–guest complexes, such as, considering flexible systems, explicit solvation, and high nuclearity systems. We focus on modelling host–guest interactions of already formed cages. However, there are examples of the impact of host–guest interactions in templating the self-assembly process.^134^

### 6.1 Describing an intrinsic cage pore

**Fig. 10:**
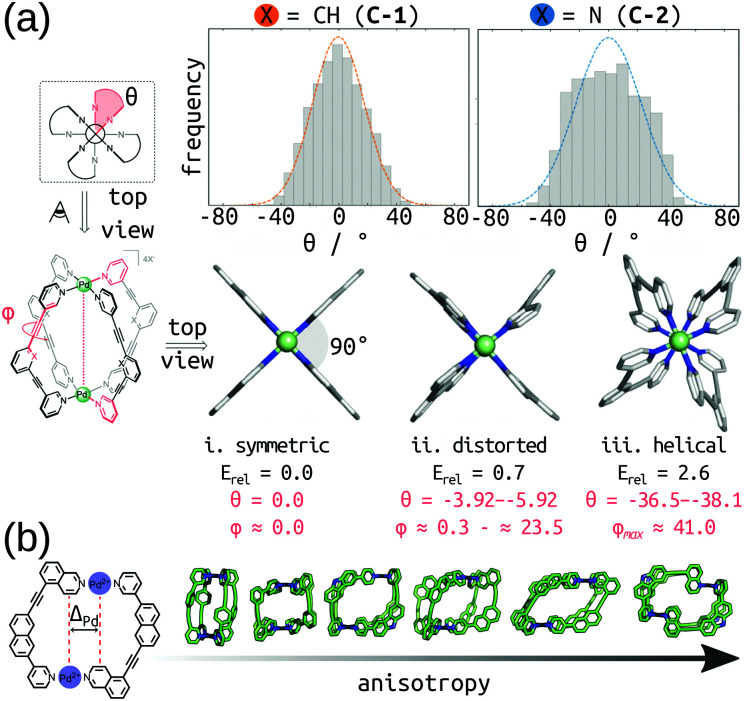
(a) (top) Twist angle (*Θ*) frequency for C-1 and C-2 calculated in explicit DCM solvent, over 30 ns of cumulative MD simulations. (bottom) local minima for C-1 (i–iii) calculated at the PBE0-D3BJ/def2-SVP level of theory. Relative energies (*E*_rel_) in kcal mol^−1^. Adapted with permission from ref. 143. Copyright 2020 American Chemical Society. (b) Geometrical measure of anisotropy (*Δ*_Pd_) used in ref. 99 with example cage structures of increasing anisotropy.

Rebek's rule provides a design rule for host–guest systems based on pore volume, where optimal binding occurs for a packing coefficient in the range of 0.55 ± 0.09.^138^ Importantly, strong intermolecular interactions or shape-effects cause deviations from this rule. The volume and shape of the pore is inherently linked to a MOCs host–guest properties. There are multiple examples of software available for analysing single-molecule pores, such as: pyWindow,^139^ MoliPor,^140^ and VOIDOO.^141^ Rizzuto *et al.* show an example of the use of VOIDOO to calculate pore volumes and help rationalise the entropic self-sorting outcome in heteroleptic MOCs.^142^ We have developed pyWindow, which uses a spherical probe for cavity measures and, like MoliPor,^140^ includes a sampling algorithm for detecting windows.^139^ Geometrical analyses of the cage and pore structure are cheap and insightful heuristics for structure–property relationships. For example, we coupled pyWindow and a geometrical measure based on the displacement of Pd centres (termed “*Δ*_Pd_” in ref. 99) to measure the size and anisotropy of the *cis*-Pd_2_L_4_ cages we generated from unsymmetrical ligands.^99^ Also, Young *et al.* measured the twist of Pd_2_L_4_ cages during MD simulations to correlate cage flexibility to guest binding affinity.^143^Fig. 10 shows example geometrical descriptors in Pd_2_L_4_ cages. Building on this, it is useful to produce energetic maps of the pore space (*e.g.*, an electrostatic potential).^101,144–146^ Finally, Rebek and co-workers asked “where do the holes in the structure end?… Where do the atoms end?”.^138^ The description of an intrinsic pore is inherently limited when assuming rigidity, and when trying to define its start and end points. Importantly, there are multiple examples of external binding sites in MOCs (*e.g.*, ref. 142 and 147). Therefore, we expect that pore representations that include the external surface and shape of the MOC will be physically useful.

### 6.2 Calculating guest binding affinity and mapping encapsulation dynamics

Calculating the binding energy of host–guest systems is an open challenge. The SAMPL blind challenges highlight multiple issues in a summary of “SAMPL7: Host–Guest challenge” including: charge, method complexity, multimeric systems, solvent/salt type and concentration.^148^ As stated in ref. 149, several variables guide the prediction of binding affinities: (1) host cavity volume, (2) guest volume, (3) ratio of guest and host volume (packing fraction), (4) involvement of solvent, (5) a combination of all of the above. All of these variables are dynamic and likely to change as a function of binding, where host–guest interactions can modify the cage structure and confinement effects. Even at the state-of-the-art quantum-mechanical level, recent work showed discrepancies between two approaches for large host–guest systems (132 atoms).^150^ DFT approaches can produce good absolute and relative comparisons to experimental free association enthalpies for relatively small organic host–guest complexes.^151^ However, quantitative insight from DFT still requires an expert touch, long compute times and some assumed conformational rigidity. Spicher *et al.* reported benchmarks for small molecule binding evaluation in MOFs, MOCs and porous organic cages using GFN*n*-xTB/GFN-FF.^152^ Importantly, the low-cost GFN*n*-xTB methods performed well throughout (including charged systems) and present generally-applicable methods. Löffler *et al.* used DFT and simplified cluster models to explore neutral guest binding (studying 50 guests) in interpenetrated Pd_2_L_4_ cages.^144^ Young *et al.* found good agreement between experimental and computed Pd_2_L_4_-quinone binding affinities when using geometries optimised at the DFT-level but found a system and geometry optimisation-method dependence on model performance.^143^ They find that the impact of the metal (Pd(ii)) in these systems mostly results in polarisation of the *ortho*-pyridine hydrogen atoms, leading to favourable quinone binding, which highlights the benefit of applying a quantum mechanical representation to the system because polarisation is not modelled in most classical approaches.

**Fig. 11:**
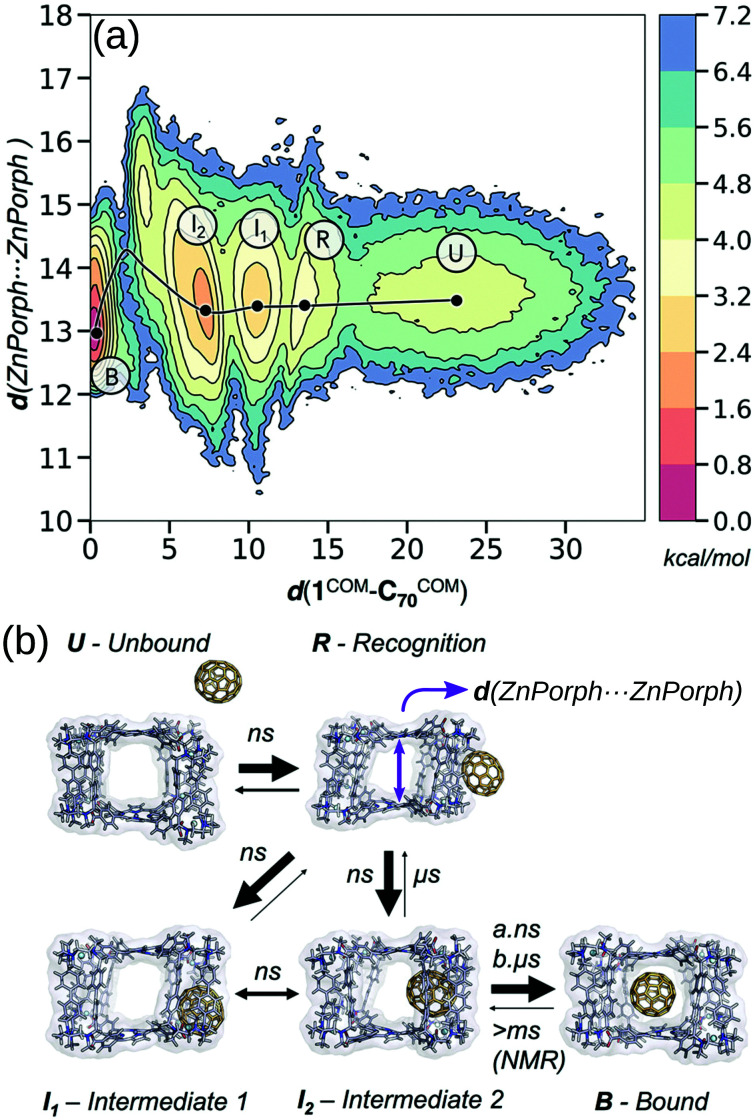
(a) Reconstructed free energy landscape of the spontaneous encapsulation process of C_70_ in the Zn and Pd-based tetragonal prismatic nanocapsule obtained from an accumulated simulation time of 75 μs of MD simulations. The landscape has been reconstructed using the host centre-of-mass to C_70_ centre-of-mass distance (*d*(1^COM^–C_70_^COM^)) and Zn⋯Zn distances (shown schematically by a purple arrow in (b)). (b) Molecular representation of five relevant metastable states: unbound (U), recognition (R), intermediate 1 (I1), intermediate 2 (I2), and bound (B) states. The width of the arrows in (b) indicates the most frequent events observed and the time scales implied from MD simulations and NMR experiments. Adapted with permission from ref. 156. Copyright 2020 American Chemical Society.

For the high-throughput evaluation of many candidates and large, dynamic systems, classical FF approaches are ideal, which has been shown continuously in their use in docking studies for protein–ligand binding. However, FF approaches are limited by chemical scope, especially for metal-containing species, and are less accurate models of nonbonded interactions (*e.g.*, lacking polarisation or context dependant parameters). Multiple recent papers^149,153–156^ explored the dynamics of encapsulation for a variety of MOCs and guests using FF approaches. In these examples, the benefit of considering dynamics of the host and guest are made clear; MOC flexibility is critical to the guest binding process, where the MOC often adapts to the guest. Classical simulations provide access to free energy landscapes, which provide structure–property relationships throughout dynamic processes. By combining NMR experiments and MD simulations, García-Simón *et al.* reconstruct the encapsulation of fullerenes into a Zn–porphyrin Pd-based MOC (Fig. 11).^156^ Additionally, the modifiable or “toy” nature of FF parameters allows for the study of implicit structure–property relationships. For example, Pesce *et al.* extracted design principles by artificially modifying the size of the guest: *e.g.*, host–guest binding energy or extent of crowding correlates with guest release rate and isomerisation rate.^154^ Importantly, each of the above papers required FF parametrisation for at least one metal species and used enhanced sampling to access the time-scales relevant to complexation.

**Fig. 12:**
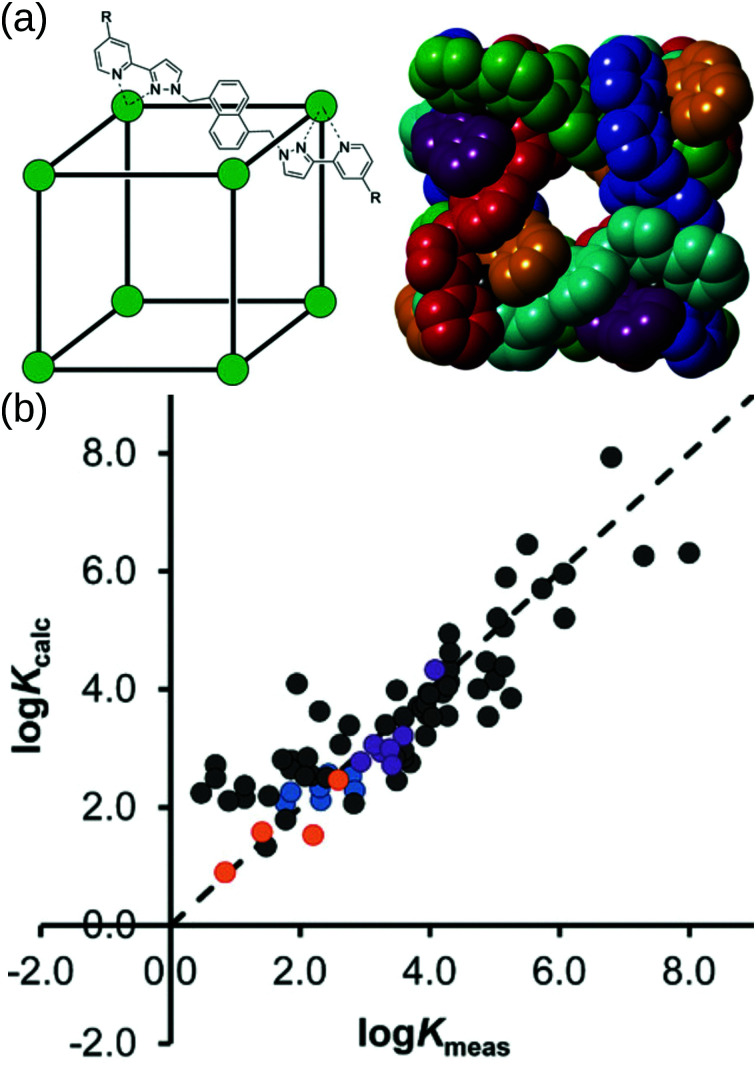
(a) (left) Sketch and (right) crystal structure of host cages Co_8_L_12_ studied in ref. 159 and 160 (R = H for crystallographic studies, R = CH_2_OH for measuring binding constants of guests in water). Comparison of experimental binding constants for the training set (*K*_expt_) with calculated binding constants (*K*_calc_) in ref. 160. Black data points are the same molecule set as in ref. 159. Orange, blue and purple data points are flexible molecules. Adapted from ref. 160.

Borrowing from the organic drug-discovery field, the Ward group^49^ implemented a docking approach to find candidate guests for their *M*_8_*L*_12_ MOC. Their capabilities hinge on the data obtained from their many experimental studies into this MOC.^157^ They initially trained a scoring function for rigid host and guest molecules on 54 data points (using the GOLD software^158^).^159^ Importantly, they only achieved good agreement when they accounted for the loss of conformational freedom due to binding in the scoring function. They support their findings by predicting (and experimentally validating) new guests from a screen of 3000 candidates. Building on this, Taylor *et al.* studied the effect of flexibility on guest binding in the same MOC.^160^ Based on new experimental data on flexible guests they retrained and improved their scoring function. Fig. 12 shows the cage structure and parity plots of the experimental *vs.* calculated binding constants from these two works. There are two limitations to this work: (1) they assume rigidity in their host complex and (2) a scoring function must be developed for each new host. However, their approach allows for the translation of protein–drug binding methods to the design of host–guest complexes with this MOC and other MOCs, given sufficient training/similar host–guest interactions.

### 6.3 Catalysis

The design of MOCs for catalysis focuses on tuning of the cavity microenvironment to optimise reaction conditions, akin to designing synthetic enzymes.^48,161,162^ The role of a MOC in catalysing a reaction is often linked to their affinity for the reactant(s), transition state, or product(s). MOC catalysis can be the result of internal or external chemistry, and/or confinement effects.^4,147,163–165^ There are many reviews on the topic of MOC-based or supramolecular catalysis,^7,8,166–168^ and *in silico* (transition metal) catalyst design.^76^ To completely capture the catalytic activity of MOCs requires detailed models (including solvent, counter/co-ions), which are costly and complex to implement. However, simplified models and heuristics for catalytic activity have been successfully uncovered, and subsequently applied. For example, through multiple computational studies into the catalytic activity of Raymond's Ga_4_L_6_ MOC,^169^ multiple design principles have been uncovered. Frushiceva *et al.* highlight the complexity of the potential paths of catalysis in this system and show the importance of the electrostatic stabilisation of the transition state.^170^ Welborn *et al.* show that the electric field, and how it changes upon confinement, provides a descriptor of catalytic activity.^171,172^ Multiple examples^173–177^ show the importance of transition-state stabilisation and solvent reorganisation under confinement to catalytic performance of this cage over multiple reactions.

Extending to multiple cage and substrate combinations, Young *et al.* developed an efficient DFT-based protocol for predicting the catalytic activity of Pd_2_L_4_ cages for Diels–Alder reactions based on quinone substrates.^143^ They begin by computationally confirming the catalytic activity of two isostructural Pd_2_L_4_ cages, explored experimentally by the Lusby group.^162^ Their protocol for calculating binding affinity and catalyic activity allows them to confirm the experimental rationalisation for the differing cage catalytic activity, highlighting the effect of sterics and flexibility. Given this rationalisation for a single reaction, they look to using their method on new substrates and testing cost-reducing assumptions toward high-throughout methods. Their approach achieves 80% accuracy with a ten-fold decrease in computation time. Coupling this with their recent work in software for metallocage construction,^101^ automated reaction-energy profile calculations^178^ and reactive FF learning (applied to Pd_2_L_4_ MOCs),^179^ they outline significant steps toward automated MOC design for catalysis. However, calculating host-based catalytic activity remains difficult to generalise to other cage/substrate systems and perform at low-cost.

## 7 Summary and outlook

We have outlined the recent progress in applying computational design processes and methods to MOCs. Much of this progress includes the development of software for tackling the structure generation and diversity (regarding the metal centre/geometry/electronic structure and the chemical diversity of organic components) of metal-containing structures. In many examples, FF/model parametrisation specific to the cage of interest was required, which limits transferability over chemical space. The development of “best-practice” guidelines for handling MOCs and metal-containing structures is pertinent to the use of design workflows in this field. An interesting approach to solving this process looks at using computational models to help design/monitor the design workflow by scoring the “safety” of calculation methods (based on DFT).^180^ Automated approaches, like ours in *stk*,^95,99^ and those of the Duarte,^101,143^ and Hay groups^80^ show progress in developing generalisable design workflows to explore MOCs. Overall, the development of low-cost cheminformatic, semiempirical and DFT approaches using large and diverse benchmark sets can provide robust and broadly applicable methods for studying MOCs with reasonable accuracy, which is crucial for high-throughput design processes.

Computational design workflows can be envisaged as feedback loops, where they are informed by and then inform design principles. Design principles are extracted by exploring chemical space and are vital for improving computational predictions. Unfortunately, many existing MOC design principles rely on a rigid picture, which quickly becomes insufficient; it is the flexibility of MOCs that provides much of their capability. Even for seemingly rigid structures, the binding of guests was shown to alter the MOC structure. Therefore, computational design must go toward dynamic pictures of MOCs while maintaining high-throughput efficiency. Advances in enhanced sampling approaches and coarse-grained models make it possible to explore potential energy surfaces of large systems, while capturing changes in structure and properties. Such a complete map can help understand the evolving and complex behaviour of functional MOCs. Additionally, the increased configurational flexibility arising from heteroleptic or anisotropic MOCs quickly results in a combinatorial explosion when attempting structure prediction. Therefore, generalisable and efficient methods serve the present and future needs of MOC chemists.

Translating tools from the solid-state materials and pharmaceuticals fields provides potential solutions. For example, we saw the power of training docking software for predicting host–guest binding affinities.^159,160^ However, this work was based on many years of experimental studies on a single cage system,^49^ which leads to a significant challenge in MOC property prediction: the collation and standardisation of experimental data for validating against. As of yet, there are no published databases of MOCs (including structures or properties) like there are for drug-like molecules and inorganic materials, which would be necessary for training robust models for property evaluation.

Providing structure and property databases allows for the application of data-science techniques and the use of machine-learning or artificial intelligence. Indeed, the flexibility of machine-learning approaches is well fit to the complexity of predicting properties of transition metals.^181^ Recent work by the Kulik group, and others, has focussed on cheminformatic-inspired approaches to transition-metal complex discovery aimed at mimicking the success of organic drug discovery platforms.^75^ Similarly, structure–property relationships can quickly find new applications for existing MOCs and help design new MOCs. Advances in artificial intelligence methods allow for the design of new molecules/materials while optimising multi-objective functions. Therefore, robust structure generation and property prediction methods are needed to allow their application to MOCs. Putting this all together will provide the generation and application of data at significantly faster rates than available in the lab and provides new insight into future design processes and decisions.

Finally, the full potential of computational tools can only be unlocked if they are usable and understandable by chemists with ranging programming or computational backgrounds. Open-source and easy-to-use software facilitates uptake by other research groups and avoids wasted development efforts, and much of our focus in software development for materials design has been to simplify the user-experience to this end. This means that rudimentary or initial calculations can be undertaken by chemists with limited computational expertise. Even where calculations do remain the domain of the expert computational chemist, the input of experimental chemists is crucial for decision-making during the process. An improved understanding of computational tools, their possibilities and limitations, helps to improve communication between collaborators. Therefore, by ensuring these steps, we expect to see more fruitful joint computational and experimental research.

## Conflicts of interest

There are no conflicts of interest to declare.

## Supplementary Material
